# A Review of Rejuvenation Strategies Targeting Mesenchymal Stem Cell Senescence and Their Impact on Bone Health and Regeneration

**DOI:** 10.1007/s11914-026-00977-y

**Published:** 2026-08-12

**Authors:** David P. Heinrichs, Rebekah M. Samsonraj

**Affiliations:** 1 Department of Biomedical Engineering, College of Engineering, University of Arkansas, Fayetteville, AR 72701 USA; 2 Division of Orthopedics, College of Medicine, University of Arkansas for Medical Sciences, 72205 Little Rock, USA

**Keywords:** Mesenchymal Stem Cells, Senescence, Bone Health, Osteoporosis, Senolytics, SASP

## Abstract

**Purpose of Review:**

The purpose of this article is to emphasize the critical role aging and senescence plays on mesenchymal stem cell (MSC) functionality and its influence on bone health. This review also highlights recent advancements in strategies to rejuvenate senescent MSCs to aid in delaying or preventing age-related illnesses associated with bone loss.

**Recent Findings:**

Studies have expanded upon many established rejuvenation strategies including senolytic agents, drugs used to selectively eliminate senescent cells, and targeted inhibitors to the secretory profile of senescent cells. New routes for MSC rejuvenation including nanoplatform targets such as biomaterials, nanovesicles, and physical exercise are showing promise for future clinical applications. Ongoing clinical trials are progressing towards rejuvenation therapies that delay age-induced bone loss.

**Summary:**

This review provides an overview of the current research landscape into rejuvenation of senescent MSCs to combat bone loss due to age and highlights new platforms and strategies for rejuvenation that could have clinical relevance.

## Introduction

Bone tissue serves a multifunctional purpose in mammals. It supports the body’s structure and motion, hematopoiesis, protects organs, and acts as a reservoir for phosphorous, calcium, and MSCs [1]. Bone health is a major indicator of age, with overall bone mineral density peaking approximately at age 30, then gradually deteriorating as age progresses. Other changes in bone due to age include increased bone resorption, inhibited bone formation, increased bone marrow fat accumulation, and multicellular senescence [2]. Bone-marrow MSCs tend to undergo adipogenic differentiation rather than osteogenesis, and overall MSC function declines. Apoptosis of osteoblasts and osteocytes increases leading to impaired bone formation, and increased osteoclast activity leads to increased bone resorption.

Due to the importance of skeletal tissue, prevention of bone density loss and functional deterioration with age is a significant and broad subject of research. Current strategies to restrict age-related bone loss from various conditions such as osteoporosis and other co-morbid conditions include rejuvenation of senescent MSCs to restore osteocyte and osteoblast functionality. This can be done through various interventions of the underlying cellular mechanisms such as senolytic treatment or secretory inhibition. Another strategy being developed to prevent age-related bone loss and rejuvenation of senescent MSCs involves lifestyle changes specifically targeting prevention of primary aging processes that subsequently provide beneficial overall effects to multiple tissues. In this review, we will explore age-related bone loss, how senescence of MSCs affects the underlying mechanisms of bone loss, and currently relevant research developments in the aforementioned strategies to prevent bone loss or rejuvenate tissue as age progresses.

Though MSC aging is characterized by multiple interconnected processes, including loss of stemness, mitochondrial dysfunction, and genomic instability, cellular senescence is a central hallmark of age-related MSC dysfunction [2, 3]. Senescent MSCs exhibit impaired proliferative and regenerative capacity and contribute to a pro-inflammatory microenvironment that negatively affects bone homeostasis and repair [4, 5]. Due to the central nature of senescence, many MSC rejuvenation strategies are designed to directly target senescence-associated pathways, while simultaneously improving other aging-related phenotypes [3]. Therefore, this review focuses on MSC senescence as a clinically relevant and well-characterized target for restoring bone health and regenerative potential.

**Fig. 1:**
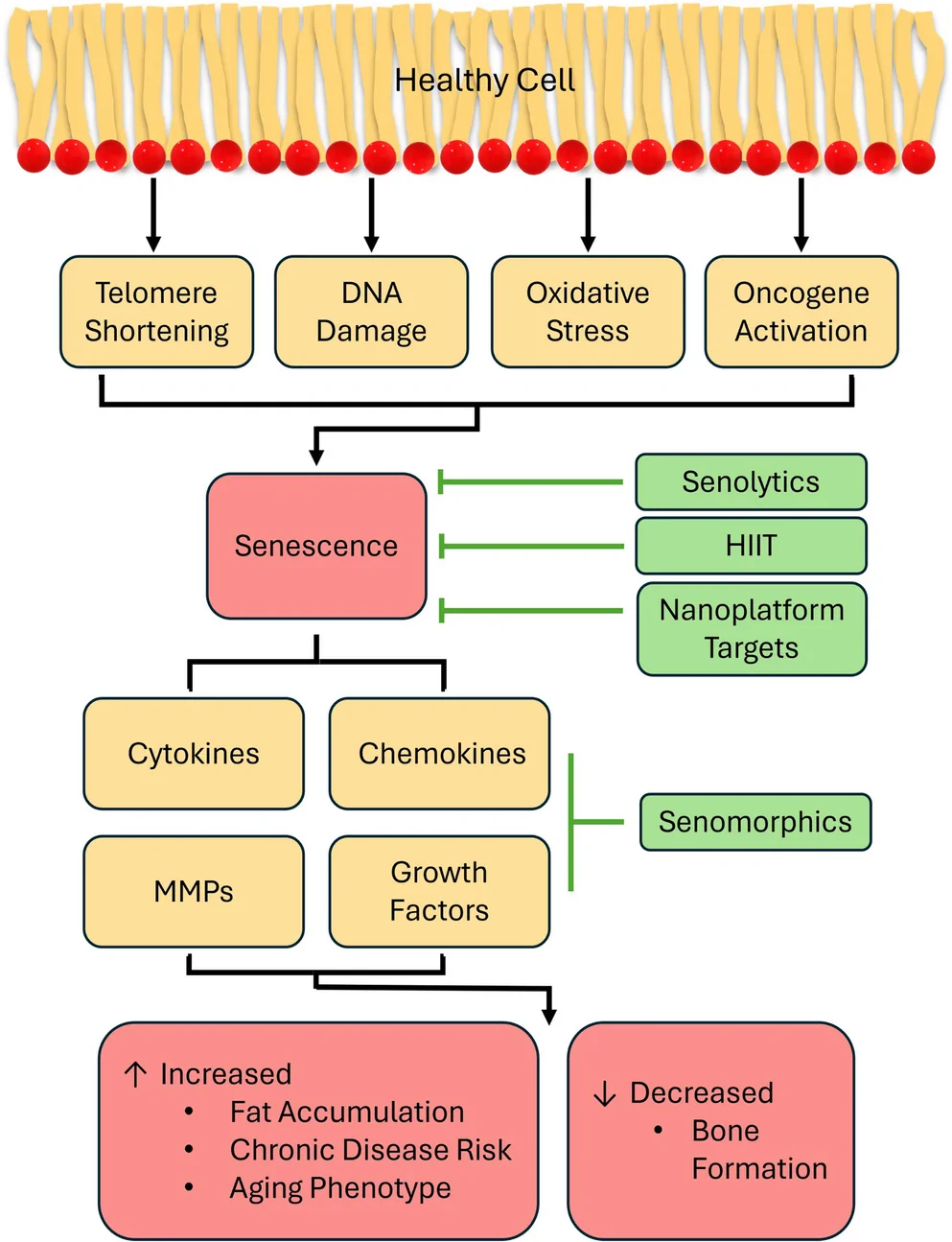
An overview of common pathways that can cause senescence in MSCs. Once cells have senesced, they exhibit a modified secretory phenotype and negative functional changes compared to normal healthy cells. The highlighted ways to combat senescence are also displayed in green boxes

## Bone Health and Aging

Normal bone healing and maintenance is a complex process involving four primary categories of cells: osteoblasts, osteoclasts, osteocytes, and stem cells. This process in adult tissue can generally be described by the following steps [7, 8]: first, osteoblasts located on the surface of the bone underneath the periosteum sense damage and begin secreting the receptor activator of nuclear factor-κβ ligand (RANKL) to recruit and activate osteoclasts to the surface of the site of damage. Osteoclasts proceed to resorb the damaged bone tissue until osteoblasts release osteoprotegerin (OPG) to bind to RANKL and block the ligand from binding to the osteoclasts; this causes the osteoclasts to stop resorption and eventually succumb to apoptosis. Osteoblasts then proceed to produce and weave osteoid to fill in the newly created void. This remodeling step can last up to months and could include vasculature remodeling, continued osteoclast-osteoblast activity, and changes to osteoid development due to external mechanical stressors. Osteocytes within the bone act as regulators of calcium and phosphate activity to control osteoblast-osteoclast remodeling to maintain homeostasis [8, 9]. They can recruit and activate osteoclasts with RANKL and are some of the longest-living cells in the body. They are most commonly differentiated from osteoblasts and trapped in the bone during remodeling. MSCs are the progenitors to osteoblasts and are recruited to the injury site via signaling of factors such as bone morphogenic protein (BMP) and TGF-β from osteoclasts [7, 10–12]. Lastly, hematopoietic stem cells (HSCs) are a critical component of the bone marrow micro-environment that continuously give rise to blood cells, immune cells, and osteoclasts [13]. They are tightly regulated by MSCs, osteocytes, and osteoblasts to maintain homeostasis in bone remodeling. This balance in resorption and reformation is disrupted during aging, which leads to chronic illnesses and generally lower bone density in older adults.

Aging heavily degrades the balance in every step of bone remodeling. Changes in the balance of bone-forming osteoblasts and bone-resorbing osteoclasts heavily biases towards bone resorption [2]. Osteoblasts form from MSCs less often and lose proliferation potential at the same time. This causes a significant decrease in OPG signaling to regulate bone degradation. Conversely, osteoclast number stays consistent or increases due to increased signaling from RANKL and other pro-inflammatory cytokines such as IL-6 and TNF-α, which greatly promotes HSC differentiation into osteoclasts. Osteoblasts show further functional losses with mechanosensing becoming impaired, RANKL secretion further increases which compounds the negative effects from osteoclasts, and increasing rates of apoptosis and senescence of osteocytes further decreases overall bone density [14]. Hormonal changes due to aging further contribute to disruption of bone remodeling, namely testosterone and estrogen modulation. Estrogen levels drastically decrease during menopause in women, and subsequently contribute to increasing levels of FSH, which is linked to increasing bone resorption [15, 16]. Testosterone also steadily decreases with age, and has as clarified association with dysfunction of bone formation in older men [15, 17]. HSCs begin to lean towards differentiation into myeloid lineages due to age and the disregulation of the homeostatic microenvironment in bone marrow [13, 18]. This functional remodeling of the bone marrow niche not only affects the ability to restore bone, but increasingly leads to chronic hematologic malignancies. Lastly, MSCs undergo various age-related changes that affect various functions in the body. For bone, MSC proliferation decreases, the differentiative capacity to form bone degrades and begins to favor angiogenesis, and MSCs can transition into a state known as senescence, a total cell cycle arrest and loss of functional potency [19–21].

### Mesenchymal Stem Cells

Of the many different cell types that make up the human body, MSCs are unique in their many functional effects. MSCs are characterized by their ability to self-renew, adhere to plastic in vitro, express the surface markers CD73, CD90, and CD105, and differentiate into mesoderm cell lineages [22]. Additionally, MSCs have been shown to secrete growth factors and other soluble molecules such as cytokines, interleukins, and chemokines that play critical roles in tissue repair and regeneration [23, 24]. In fact, the minimal criteria to define MSCs has been greatly expanded in recent years to include other factors such as the production of other novel trophic factors, and a defined mRNA profile [25]. All these factors together make MSCs ideal candidates for therapeutic applications. MSCs play a critical role in normal bone regeneration and healing as well. They are the progenitor cells for osteogenic cells and subsequently osteoblasts through a process called osteogenesis, making them a critical pillar in all bone healing and remodeling [6, 26].

MSCs within the skeletal system are increasingly recognized as heterogeneous populations that reside and function in distinct anatomical niches. Bone marrow-derived MSCs exhibit distinct subpopulations within their own niche to maintain stemness, proliferation, and function [27–29]. Aside from bone marrow-derived MSCs, skeletal stem/proginitor cells have been discovered in the periosteum, endosteum, and perivascular regions surrounding bone. These cells’ fates are decided by environmental cues and have been found to be crucial for maintaining homeostasis and fracture repair via osteochondral differentiation [30, 31]. Emerging evidence further suggests that these progenitor populations respond differently to aging, injury, and disease-associated stimuli. Age-related skeletal disorders such as osteoporosis and impaired fracture healing likely arise from dysfunction across multiple progenitor niches [32, 33]. Though the term MSC is used throughout this review, it is important to recognize that skeletal MSCs comprise diverse populations with distinct biological functions and regenerative capacities.

### MSC Aging and Senescence

 As previously discussed, stem cell aging is a multifaceted cascade of various stressors and alterations that lead to a loss of stemness, and ultimately, senescence. This major obstacle for using MSCs for cellular therapy is characterized by an irreversible replicative halt within the cell cycle that causes resistance to apoptosis and increased metabolic activity [5, 34, 35]. The main drivers of aging and senescence (Fig. 1) are cellular replication and oxidative stress [35, 36]. Replicative senescence was first observed in the 1960s and the term “Hayflick limit” was coined to denote the proliferative cessation observed in fibroblasts upon reaching a certain number of population doublings [34]. This limit is reached due to telomere shortening during cellular replication, where the terminal 50–200 base pairs of a linear chromosome are not copied during lagging strand DNA replication [37]. Telomeres continue to shorten until they reach a critical length (around 10 kilobase pairs [38]) which triggers a DNA damage response that pushes a cell towards replicative senescence. Oxidative stressors cause the DNA damage response to trigger prematurely in cells, dramatically shortening the time for cells to hit the Hayflick limit. Reactive oxygen species (ROS) such as hydrogen peroxide and superoxide anions are naturally occurring in the body, though cells normally have antioxidant defense mechanisms in place to minimize the stress they cause. However, ROS can be overproduced in the body, or impaired antioxidant mechanisms can effectively increase the oxidative stress on cells, leading to DNA damage, and in turn, replicative senescence [39]. These stressors and the senescence they cause all lead to an overall decline in potency for MSCs. Various other age-related functional changes can be observed leading up to cellular senescence. Aging cells tend to be much larger than young proliferating cells, which could indicate abnormal biosynthesis or cell cycle progression [5, 40]. They also exhibit impaired migration and homing abilities, which are both crucial for bone remodeling [41]. Most importantly, aged MSCs skew their differentiative capacity towards adipogenesis over osteogenesis or progressively lose their ability to differentiate entirely [41–43]. 

Senescent MSCs contribute to the degeneration of the bone microenvironment in various ways. As previously discussed, their impaired differentiative capacity leads MSCs towards adipogenesis or complete dysfunction of differentiation completely. Impaired paracrine signaling of senescent MSCs can increasingly cause secretion of interleukins and ROS that damage adjacent cells and subsequently, induce senescence [4, 39, 44]. RANKL and OPG signaling that regulates osteoclast function is disturbed, leading to increased osteoclastogenesis [2]. Normally functioning MSCs secrete angiogenic factors such as VEGF and HGF, and senescent cells often exhibit reduced production of these factors [40, 45]. This leads to reduced vascularization, and in turn, slower healing and impaired fracture repair. MSCs also function normally as immunoregulatory cells, suppressing T-cell activation and promoting anti-inflammatory M2 macrophages. The SASP degrades normal immunosuppression by reducing normal suppression signals such as IDO and actively secreting signals such as CCL2 and CXCL10 that recruit T-cells [46]. Compounded with reduced immune surveillance due to age, this signaling can lead to chronic inflammation. Together, these dysfunctions of senescent cells directly affect homeostasis of the bone microenvironment, leading to an overall decline in remodeling and fracture healing. Senescence has some beneficial effects in the cell cycle such as being an anti-cancer mechanism, and assisting in wound healing [20], though ideally, immune cells would clear senescent cells through apoptosis after they had outlived their usefulness. However, senescent cells can easily avoid immune clearance, leading to detrimental effects when allowed to persist in tissues for prolonged periods [19, 47]. To effectively investigate and combat persistent senescence of MSCs, researchers have investigated various ways to target, destroy senescent cells, and prevent further senescent cells from developing.

## Standard Bone Healing Enhancers and Systems Biology

Though MSCs play a major role in bone healing, and rejuvenation of MSCs is a promising route for researchers to enhance the healing process, it is beneficial to introduce some standard methods clinicians use to enhance bone healing. Bone morphogenic proteins (BMPs) have been the most well characterized and widely studied growth factors for bone regeneration for over 20 years. The canonical pathway of BMP signaling involves binding to cell surface receptors, leading to a complex formation composed of two dimers of type I and II serine/threonine kinase receptors, eventually resulting in activation of the Smad pathway [48, 49]. The FDA has approved rhBMP-2 for various treatments such as open tibial shaft fractures and anterior lumbar interbody fusions[50]. Another common treatment for fracture repair used in the clinic is bone grafting. These grafts are often performed during surgeries to improve post-operative healing, and the platform encompasses allogenic, autologous, xenogenic, and even synthetic grafts [51]. Bone grafts make up the second-most transplanted tissue in the world after blood, though they come with many complications and risks including site infection, general anesthetic, pain, or secondary fractures [51–53]. Standard enhancements to bone healing extend beyond fracture-specific therapies and, more relevant to this review, cover age-related conditions such as osteoporosis. Common anti-osteoporotic therapies include anabolic agents, anti-resorptive agents, and optimized nutrition [54]. Anabolic agents act to promote bone formation via activation of the human parathyroid hormone receptor and subsequently, increase serum calcium levels. Currently approved anabolic agents for osteoarthritic patients include teriparatide and abaloparatide [55]. Anti-resorptive agents cover a variety of drugs including bisphosphonates, RANKL inhibitors, and estrogen regulators, though they all act to regulate osteoclast activity to mitigate bone resorption [54, 56–58].

Outside of MSC-mediated recovery of osteocytes, there is evidence that skeletal rejuvenation may also in part be influenced by immune regulation, rejuvenated angiocrine factors that release from organ-specific endotheliums, and inter-organ crosstalk [59, 60]. Post-menopausal osteoprorosis is a prime example of the systematic nature of bone degeneration. It increasingly presents as a network disorder in which estrogen deficiency and aging‐related immunometabolic drift couple skeletal fragility to sarcopenia, adipose inflammation, gut dysbiosis, vascular dysfunction, and neurobehavioral changes that collectively shape a frailty phenotype [61]. This frailty phenotype may be treated with a multifaceted approach that targets multiple parts of the system to improve outcomes on bone remodeling. One example of this methodology is a recent study that augmented the lymphatic system in mice to improve digit regeneration in a digit amputation model [62]. The researchers found that impairment of the lymphatic vessels at the side of amputation lead to increased osteoclast function to accelerate clearance of damaged tissue and bone, and thus, improved subsequent digit regeneration. Although systemic interventions targeting immune regulation, vascular function, and inter-organ communication have demonstrated regenerative potential, accumulating evidence suggests that many of these pathways converge on the regulation of MSC function, thereby influencing skeletal repair and regeneration [59, 60, 63]. Consequently, MSC rejuvenation represents a critical focal point for understanding and enhancing age-related bone regeneration. The following sections examine current strategies for reversing MSC aging and restoring their regenerative capacity.

## Rejuvenation of MSCs to Promote Bone Formation

### Senolytic Agents

The class of drugs known as senolytics were first discovered over a decade ago by the Mayo Clinic Center on Aging [3]. These first-generation senolytics were identified via observations of how senescent cells potentially resisted apoptosis through the Senescent Cell Anti-Apoptotic Pathway (SCAP). They used RNA interference to find senescent cells depending on this pathway to evade apoptosis then used bioinformatics to find compounds whose mechanisms of action targeted this SCAP network, focusing on translating compounds already in use for other conditions. The most widely studied senolytics from these first-generation compounds on MSCs include dasatinib (D), quercetin (Q), fisetin (F), and curcumin (C) (Table 1).

**Table 1:** A list of the highlighted senolytic and senomorphic agents discussed in this review with notable references for more information

Name	In-text Abbreviation	Description	Notable references
Dasatinib	D	Tyrosine kinase inhibitor, used to treat leukemia	[64, 65, 79, 80, 85–87]
Quercetin	Q	Antioxidant flavanoid	[68–70, 85–87]
Fisetin	F	Antioxidant flavanoid	[69, 72, 83, 91]
Curcumin	C	Polyphenol antioxidant	[75–78]
Losartan	L	Angiotensin receptor blocker	[90, 91]
BIO1	N/A	Novel senolytic, upregulates p53	[92, 93]
Phytic Acid	N/A	Nutritional antioxidant that inhibits mineral absorption	[94–96]
Metformin	N/A	Type 2 diabetes medication – decreases glucose production	[102–107]
Rapamycin	N/A	mTOR pathway inhibitor, antibiotic used in transplants	[110, 111]
Epigallocatechin gallate	EGCG	Antioxidant flavanoid	[112–114]
AG490	N/A	Tyrosine kinase inhibitor, JAK2/3 inhibitor	[115]

D was used primarily as a treatment for myeloid leukemia and gastric cancer [64, 65] until it was identified as a potential senolytic agent. D is a src/tyrosine kinase inhibitor that promotes apoptosis through interference of multiple parts of the ephrin survival-regulating dependence receptors (ENFB) [3, 42, 66, 67]. Q is a compound in the flavanoid family, commonly found in citrus fruits, with many beneficial effects such as antioxidant protection and regulation of cell growth [68]. The pro-apoptotic effect of Q on senescent MSCs is still being studied, but currently it is known to primarily affect the Hippo signaling pathway, more specifically the YAP/TAZ transcriptional co-activators [69, 70]. Q has been shown to promote YAP/TAZ expression to improve both stemness and osteogenic capacity [71]. F is another compound in the flavanoid family identified as a potential senolytic. Its structure is similar to Q, however it has different biological effects on the same signaling pathways to its flavanoid cousin [70, 72]. Both of the flavanoid senolytics are also known to affect the anti-apoptotic Bcl-2 family proteins, upregulating and downregulating pro-apoptotic proteins and damaging the mitochondrial transmembrane potential in MSCs and cancer cells [3, 73, 74]. Finally, C is a polyphenolic compound found in turmeric as the active ingredient. C is one of the most potent antioxidants, and it is known to activate the nuclear factor-erythroid-2-related factor 2 (Nrf2) pathway which has a pivotal role in activating antioxidative enzymes [75, 76]. The senescence potential of C is due to its ability to activate and modulate autophagy pathways in a cell, preventing or reversing senescence altogether [77, 78]. These senolytics are optimal targets for research as they both promote apoptosis of senescent cells and have low off-target effects on non-senescent cells.

Research into amelioration of senescent MSCs through senolytic interventions has greatly progressed in the last five years for the previously described first-generation senolytics. Research has further discovered novel senolytics to rejuvenate senescent MSC populations to improve bone remodeling. Individual assessments of D have shown improved mineralization of MSCs and significant increases in osteogenic differentiation markers SP7, ALP, and COL1A1 [79, 80] in older populations of MSCs compared to younger actively proliferating groups [42]. When tested individually, Q shows varied results with some recent published results displaying little to no effect to bone rejuvenation of treated senescent populations, and others providing evidence supporting its rejuvenation capacity [43, 81]. Investigations of F show further variable results, with a systematic review finding increased osteoblast formation and reduced osteoclast production via activation of the PI3K-AKT pathway, upregulation of the transcriptional activity of Runx2, and maintenance of calcium and phosphorus levels in vitro and in vivo [82]. Another study on F’s effect on pre-osteoblasts in vitro and in vivo in an accelerated aging mouse model showed significant reduction to senescence-associated transcriptional factors p16, IL-6, and TGF-β while attenuating bone loss in vivo [83]. A contradictory study provides evidence showing that F inhibits proliferation and migration of MSCs, inhibits osteogenic differentiation via inhibition of the YAP signaling pathway in MSCs, reduces expression of osteogenic markers COL1A1 and ALP, and potentially causes dedifferentiation of MSCs towards a more primitive fibroblast-like state [70, 72]. Research development investigating C has focused on discovering the signaling pathways C modulates and how C effects MSC rejuvenation as a senolytic. Researchers have determined that C modulates senescence via activation of autophagy pathways including FOXO3 and promotes lysosomal acidification [77, 78]. Further, evidence of amelioration of senescent populations through activation of mitophagy was presented through liposomal delivery of C in bone-marrow derived MSCs [76]. Induction of C-treated adipose-derived MSCs in vivo appears to enhance the therapeutic effects on cartilage degradation, synovial inflammation, and bone deterioration in osteoarthritic mice [78].

Progressive studies into senolytic therapies have begun testing combinations of senolytics, with the most widely studied combination being D and Q. DQ treatment has been widely studied since 2015 [84] on various age related conditions [86, 88], and it is reported to have a synergistic effect compared to using singular senolytics [71, 87]. Recent reports show combination DQ treatment effects the YAP and TAZ networks to promote stemness maintenance and improve osteogenesis regardless of senescence [71]. In vivo studies have begun to become standard in recent years, with evidence of combination DQ retarding bone degeneration in post-menopausal osteoporotic mice [87]. Another group has shown improvements to wound healing with biomaterial implants when DQ is taken orally due to the elimination of stress-induced premature senescent cells [88]. Another group has shown improvements to bone regeneration through direct implantation of DQ-treated MSCs in a calvarial defect [89]. Aside from combination DQ, researchers are beginning to explore other combinations of senolytics such as F and losartan (L) (another potential senolytic first reported in 2010 [90]). This combination study showed FL reduced inflammation and senescence markers IL-1β and IL-6, and improved osteogenic potential in vitro without impacting adipogenesis [91].

Novel senolytic agents have also been developed in recent years with promising early results on skeletal muscle and MSC rejuvenation. One senolytic known as BIO1 inhibited MDM2 binding to the p53 gene, causing improvements to muscle regeneration and hypertrophy in mice, and reduced the abundance of senescent mononuclear cells in muscle [92]. The same group further found BIO1 rejuvenates resident cells and significantly decelerates DNA methylation with age [93]. This promising work could have prospects with further research into the relationship between remodeled skeletal muscle and bone degeneration with age. An example of a compound being novelly repurposed for senolytic purposes is phytic acid, an organic phosphoric acid typically used as a surface modification for implants to improve MSC proliferation and osteogenic differentiation [94, 95]. New research has provided evidence showing phytic acid as a potential senolytic, its ability to attenuate decreased osteogenic potential in a high glucose environment, and that it inhibits MSC senescence through the ERK signaling pathway [96]. These new developments in rejuvenation of senescent cell groups provide a more complete picture of the attenuation of bone degeneration with age and provide a great breadth of evidence for future clinical practices.

## SASP Modulation

Aside from senescent cells’ own effects on surrounding tissue, the secretome, or SASP, of senescent cells actively influences the micro-environment of surrounding cells and tissues. The SASP is a crucial change in the secretory profile of senescent cells versus their non-senescent counterparts that influences stem cell function, immune responses, and extracellular matrix balance, making it an ideal target for MSC rejuvenation and enhancing tissue repair [97]. The SASP varies widely across cell types but generally comprises an array of cytokines (e.g. IL-6, TNF-α), chemokines (e.g. CCL2), growth factors (e.g. VEGF, HGF), matrix metalloproteinases (MMPs) and extracellular vesicles (EVs) [97–99]. MSCs secrete a multitude of specialized SASP proteins that are largely donor dependent, including ACTα2, COL1A1, and SOD1, of which are especially relevant to bone aging [4]. Attenuation of the SASP falls under two main categories: compounds that clear senescent cells such as the previously discussed senolytic agents, and direct inhibitors of secretory pathways known as senomorphics [100]. Studies into rejuvenation of senescent cells via senolytic agents normally include evidence of downregulating common SASP factors [77, 87, 91, 101] such as the aforementioned cytokines, chemokines, and MMPs, so we will pivot towards senomorphic interventions.

The SASP is governed by various signaling pathways such as NF-κβ, mTOR signaling, and the JAK/STAT signaling pathway. Senomorphic agents target the SASP mainly through inhibition of these signaling cascades to neutralize the activity and function of the SASP components [100]. Many of these SASP inhibitors have been discovered or developed throughout the last two decades, but more recent developments on MSC-specific rejuvenation have been focused on a select few senomorphic agents. For example, metformin is an FDA-approved medication used to treat type 2 diabetes that has begun to be repurposed for its NF-κβ inhibitory effects [102]. In the last five years, metformin has been shown to restore signaling pathways associated with normal MSC functions [103], rejuvenate migration and T cell regulatory properties of bone-marrow MSCs [104], and promote osteogenic differentiation via the AMPK/BMP/Smad pathway [105–108]. Senomorphics also appear as natural compounds like the flavanoid senolytics. Rapamycin is an antibiotic mainly used to prevent transplant rejection by graft-versus-host disease [109], and it too has shown senescence rejuvenation of MSCs via inhibition of the mTOR signaling pathway [110], increased autophagy properties, and increased osteogenic and chondrogenic differentiation in MSCs [109, 111]. Epigallocatechin gallate (EGCG), an extract from green tea, effectively inhibits SASP components via activation of the SIRT3/NRF2 pathway [112, 113] and restores bone formation in vivo in a senomorphic conjugated vacuum-heated gelatin sponge scaffold [114]. Lastly, novel studies into JAK/STAT3 inhibitors such as AG490 have begun to progress to inhibit senescence in MSCs and promote therapeutic effects for conditions such as liver fibrosis [115] and may be translated for osteogenic rejuvenation in the future.

### Nanoplatform Targets

Rejuvenation has extended beyond senolytic and senomorphic agents in recent years. Research groups have begun to utilize nanoplatform targets, namely EVs, to restore the therapeutic potential in senescent MSCs. As previously discussed, utilizing liposomes to deliver the senolytic C to MSCs improved the proliferation and mobility of senescent cells, activated mitophagy via specialized targeting, and can potentially improve the general stability and bioavailability of C in vivo [76]. Another group has recently tested coated liposomes loaded with Q to specifically target senolytic delivery to bone [116]. The liposomes were coated with a bone affinity peptide and loaded with Q. Administration in vivo effectively eliminated senescent MSCs in bone and restored functional osteogenesis. Further than senolytic delivery, exosomes derived from embryonic stem cells (ESCs) are also beginning to show promise for age-related cell and tissue rejuvenation, first shown with mouse ulcers [117]. This has been developed in conjunction with exosomes isolated from MSCs delivered to senescent cultures to ameliorate senescence in various cell types such as osteoarthritic osteoblasts, MSCs for myocardial repair, tendons, and ovarian function [118–122]. This platform appears to work via the various miRNAs carried by EVs for paracrine signaling (e.g. miR-21-5p, miR-17-5p, and miR-199a-3p) [121, 123, 124] though it has not been established which specific growth factors or miRNAs play a targeted therapeutic role. MSC-derived EV rejuvenation has recently been shown to rejuvenate MSCs to improve migration and proliferation, enhance autophagy, increase osteogenic differentiation, and enhance new bone formation in rats [122]. Exosomal rejuvenation of senescent MSCs shows great promise for future therapies as a simple and easily expandable platform to rejuvenate aged MSCs and may have potential to open the door for unrestricted allogenic MSC transplantation in the future for aged patients [119].

Nanoplatform rejuvenation strategies extend beyond EVs and nanovesicle drug delivery. Many biomaterial-based strategies have been developed in recent years that exhibit anti-senescent properties by tweaking the physical cues cells experience during in vitro expansion. One group has recently developed a 2D nanotopogrpahical substrate that can be used to study MSCs under varied surface modifications compared to a normal flat substrate. The group found significant changes to cytoskeletal and nuclear envelope structure, as well as many other phenotypic and genotypic changes that have potential implications for senescence rejuvenation [125]. In a review discussing this article, the authors highlight the potential of nanopit topography to regulate MSCs morphology and potentially reverse senescence via physical cues and suggest that biomaterial surface modifications could modulate the differentiation potential of senescent MSCs through mechanical regulations [126]. Another group has found that Osteopontin (OPN), a glycosylated phosphoprotein and a key extracellular matrix molecule in bone tissue, can be used as a material surface coating to rejuvenate senescent MSCs [127]. When OPN was coated on generic cell culture plates, senescent MSCs began to exhibit a rejuvenated genetic profile and restored osteogenic capacity. MSCs seeded on the OPN coating also appeared to change morphology from a flattened wide shape, to an elongated fibroblast-like shape analogous to non-senescent cells. These studies indicate the influence that physical cues have on senescent MSC populations and their potential senotherapeutic effects. 

One final nanoplatform target that has been discovered recently is the first senescent MSC-specific surface marker that has been used to rejuvenate bone repair via an active targeting platform [128]. This platform uses a senescence-targeted and NAD+-dependent SIRT1-activated micelle, engineered to promote aged bone regeneration. The micelle is loaded with resveratrol and NAD^+^ boosters to promote activation of the SIRT1 pathway and targets senescent MSCs via antigen binding of Kremen1, a newly identified senescent cell-specific surface marker. This novel approach shows increased proliferation and improved osteogenic potential in vitro and in vivo when the platform is loaded into a hydrogel and implanted into a bone defect [128]. This is an important study to highlight as the first reported active targeted antigen binding senescent cell-specific rejuvenation strategy and can open a plethora of new rejuvenation strategies for MSC therapies in future research.

## Exercise Rejuvenation

A 2021 systematic review of various exercise investigations first brought up the question of exercise’s potentially senolytics effects on MSCs [129]. The study broadly defined a senolytic effect as a decrease in p16^INK4A^, senescence-associated beta galactosidase (SAβgal), or p21^Cip1^ and concluded that various forms of exercise in both animals and humans significantly reduced p16^INK4A^ levels for senescence maintenance in various cell types, while more heterogeneous results were observed for p21^Cip1^ and SAβgal expression. To provide more evidence of the underlying pathways that govern exercise rejuvenation, a recent study found that exercise regulates the osteogenic differentiation of MSCs through the Antxr1/LncRNA H19/Wnt/β-catenin axis, and in the study, found that exercise alleviated bone loss due to ovariectomy in mice [130]. Studies have also found that exercise dramatically increases HSC generation in the bone marrow niche, which could help regulate the skeletal micro-environment [131]. Finally, a very recent study sought to identify the effects of exercise in rejuvenating MSCs and explore associated molecular network mechanisms [132]. Their results suggest that exercise exerts its beneficial effects on MSCs primarily through metabolic and inflammatory signaling pathways. They concluded that rejuvenation via exercise specifically involves inhibition of IL-1β, IL-6, p16, and p21. 

More developments in the research landscape have emerged showing the potential of physical exercise to combat senescence, and more notably, rejuvenate bone mineral density and muscle strength in aged subjects [133–135]. An in vitro study showed that mechanical stretch inflicted on MSCs reduced common senescence markers and improved osteogenesis via upregulation of CXCR4, a key upstream regulator in the PI3K/AKT signaling cascade [136]. In vivo research has shown that running promoted autophagy via activation of SIRT1 and significantly alleviated bone loss in aged mice [137]. Exercise further may affect bone remodeling through rejuvenation of muscle cells. Interactions between muscle and bone were historically assumed to be solely through mechanical coupling, though today it is recognized that muscle and bone act as endocrine organs, producing and releasing EVs and osteokines to influence each other’s functions. These interactions have been coined “bone-muscle crosstalk,” and there are numerous studies showing muscle’s influence on bone, especially in the context of aging and rejuvenation [138–140]. A human study showed the rejuvenating effect of high intensity interval exercise on senescent stem cells located in muscle fibers [141], and another in vivo mouse study showed that late-life resistance training improved molecular, biochemical, and cellular function in skeletal muscle including ribosomal and mitoribosomal protein regulation by DNA methylation [142]. Overall, research shows that exercise, specifically high intensity interval exercise and running [137, 141], provides a significant senolytic effect to rejuvenate MSCs in bone and muscle and improve outcomes of aging subjects. 

Another primary detrimental effect in aging cells is the creation of damaging ROS in the mitochondria [143, 144]. Damaged mitochondrial DNA accumulates more with age and exacerbates ROS production. Mitochondria also act as a driver of differentiation in MSCs, making metabolic rejuvenation an ideal target for therapy. Studies have found that exercise has dramatic effects on mitochondrial quantity, function, and quality. In mice, researchers have found notable increases in transcription factors that control the expression of several bioenergetic pathways, restoration of age-related deficits in autophagy, and reduction of mitochondrial DNA mutations after various forms of short- and long-term exercise [145]. More recent study has been performed on human subjects, corroborating improvements to metabolic activity and insulin resistance with long-term exercise [135].

## Current Status of Clinical Trials

Currently, there are several clinical trials investigating rejuvenation of MSCs to improve bone health with age. One trial (ID: NCT03169231) began in 2017 to infuse increasing amounts (25 million – 200 million) of allogenic MSCs into 70–85 year old patients to combat aging frailty using a 6-minute walk test (6MWT). The primary outcome is a change in 6MWT compared to placebo at 180 days and secondary outcomes include changes in patient reported outcomes of overall physical function capacity and a change in serum TNF-α compared to placebo. This trial is currently in phase 2b as of 2026. A second trial (ID: NCT06018467) began in 2023, seeking to implement dietary use of the senolytic drugs D (100 mg/day for 2 days) and Q (1.25 mg/day for 3 days) in patients aged 60–90 with increased fracture risk to improve osteoporosis therapy. The primary outcome is measured by a change in circulating marker of bone resorption (CTX) measured at baseline and at week 21 and secondary outcomes include a change in circulating marker of bone resorption Tartrate Resistant Acid Phosphatase (TRAcP) at 21 weeks and a change in circulating markers of bone formation (PINP, osteocalcin, and bone alkaline phosphatase) at 21 weeks. This trial is currently in phase 2 as of 2026. Many other therapies are in the pre-clinical phase, but the current clinical research shows great progress for clinical use of rejuvenation therapies in the future to help combat age-related bone loss conditions.

### Conclusions and Broader Effects

A majority of the outcomes discussed by the researchers in these articles have included osteogenic differentiation or some degree of bone-oriented protein, DNA, or growth measurement. However, many of the discussed treatments improve other aspects of MSC functions that can contribute to skeletal repair. Some previously discussed examples include SASP modulation limiting negative effects to neighboring cells, rejuvenation of immunosuppression to mitigate local chronic inflammation around bone fractures, and pro-angiogenic factors improving bone micro-environment homeostasis along with HSCs. Overall, these examples suggest that the therapeutic benefits of MSC rejuvenation extend beyond direct osteoblast formation, contributing to bone healing through multiple complementary mechanisms.

The search for the key to rejuvenation of senescent MSCs to target age-related conditions is continually expanding. Some focus of research is digging deeper: such as signaling pathways, targeting specific effects of known senolytics and senomorphics, and pursuing in vivo and clinical studies for the most promising current therapies. Other research is focused on broadening the horizons of rejuvenation such as discovering new potential senolytic and senomorphic agents, repackaging senolytics in nanoplatforms, or identifying and targeting novel antigen binding platforms. While there are concerns about off-target effects and side effects due to the rapid cell clearing effects of senolytics, there is great promise in current translational medicines and clinical trials as the agents are currently FDA-approved for use. With ongoing clinical trials, and new targets emerging from preclinical studies, the coming years are likely to reveal more novel MSC rejuvenation strategies to ameliorate bone degeneration along with other age-related conditions 

## Data Availability

No datasets were generated or analysed during the current study.
